# Confirmation of the predictive function of cuproptosis-related gene FDX1 in clear cell renal carcinoma using qRT-PCR and western blotting

**DOI:** 10.18632/aging.204807

**Published:** 2023-07-10

**Authors:** Congbo Cai, Kena Zhou, Jing Jing, Yu Ren, Guobin Weng, Dong Cen, Xue Wang, Shuaishuai Huang

**Affiliations:** 1 Department of Emergency, Ningbo Urology and Nephrology Hospital, Ningbo Yinzhou No.2 Hospital, Ningbo 315100, Zhejiang, China; 2 Shanghai Jiao Tong University School of Medicine, Shanghai 200025, China; 3 Department of Laboratory, Ningbo Urology and Nephrology Hospital, Ningbo Yinzhou No.2 Hospital, Ningbo 315100, Zhejiang, China; 4 Department of Ultrasound, Ningbo Urology and Nephrology Hospital, Ningbo Yinzhou No.2 Hospital, Ningbo 315100, Zhejiang, China

**Keywords:** FDX1, curproptosis, clear cell renal carcinoma, immune response, immunotherapy

## Abstract

Background: Cuproptosis is a novel cell death mechanism, and FDX1 is a key gene associated with cuproptosis. However, it is unclear whether FDX1 has prognostic and immunotherapeutic value for clear cell renal carcinoma (ccRCC).

Methods: Data on FDX1 expression in ccRCC were extracted from various databases and validated using qRT-PCR and western blotting. Moreover, the survival prognosis, clinical features, methylation, and biological functions of FDX1 were evaluated, and the tumor immune dysfunction and exclusion (TIDE) score was used to explore the immunotherapy response to FDX1 in ccRCC.

Results: The expression of FDX1 in ccRCC tissues was significantly lower than that in normal tissues, as validated by qRT-PCR and western blotting of patient samples (*P* < 0.01). Moreover, low FDX1 expression was related to shorter survival time and high immune activation, as indicated by alterations in the tumor mutational burden and tumor microenvironment, stronger immune cell infiltration and immunosuppression point expression, and a higher TIDE score.

Conclusions: FDX1 could serve as a novel and accessible biomarker for predicting survival prognosis, tumor immune landscape, and immune responses in ccRCC.

## INTRODUCTION

According to the GLOBOCAN report, an estimated 431,288 people are diagnosed with renal carcinoma each year, accounting for 2.2% of newly discovered cancer cases [1]. The most common histological subtype of renal carcinoma, clear cell renal carcinoma (ccRCC), accounts for 80–90% of all cases [2]. Early ccRCC has a favorable prognosis, whereas advanced ccRCC can result in considerable recurrence and high mortality. Nevertheless, targeted therapy and immunotherapy have altered the treatment patterns of advanced ccRCC [3]. Immune checkpoint inhibitors (ICIs) have been proved to be an important and effective strategy in the therapy of ccRCC [4, 5].

FDX1 is an important regulator of copper ionophore-induced cell death [6, 7]. Previous studies have linked FDX1 to a variety of cancers, including lung adenocarcinoma [8], ccRCC [9], hepatocellular carcinoma [10], and colon adenocarcinoma [11]. FDX1 plays a critical role in tumor occurrence, development, prognosis, and treatment [8]. Researchers have proposed that FDX1 is associated with ccRCC prognosis [12], but its value in immunotherapy remains unknown.

Using The Cancer Genome Atlas (TCGA) database, we investigated the expression, prognostic value, biological function, methylation, and protein transcription of FDX1 in ccRCC. We focused on evaluating the significance of FDX1 in immunotherapy, while validating the results using independent datasets from external databases. In addition, 75 patients with ccRCC were recruited from the Ningbo Urology and Nephrology Hospital (NBUNH) for experimental and clinical verifications.

## RESULTS

### Expression of FDX1

**Figure 1:**
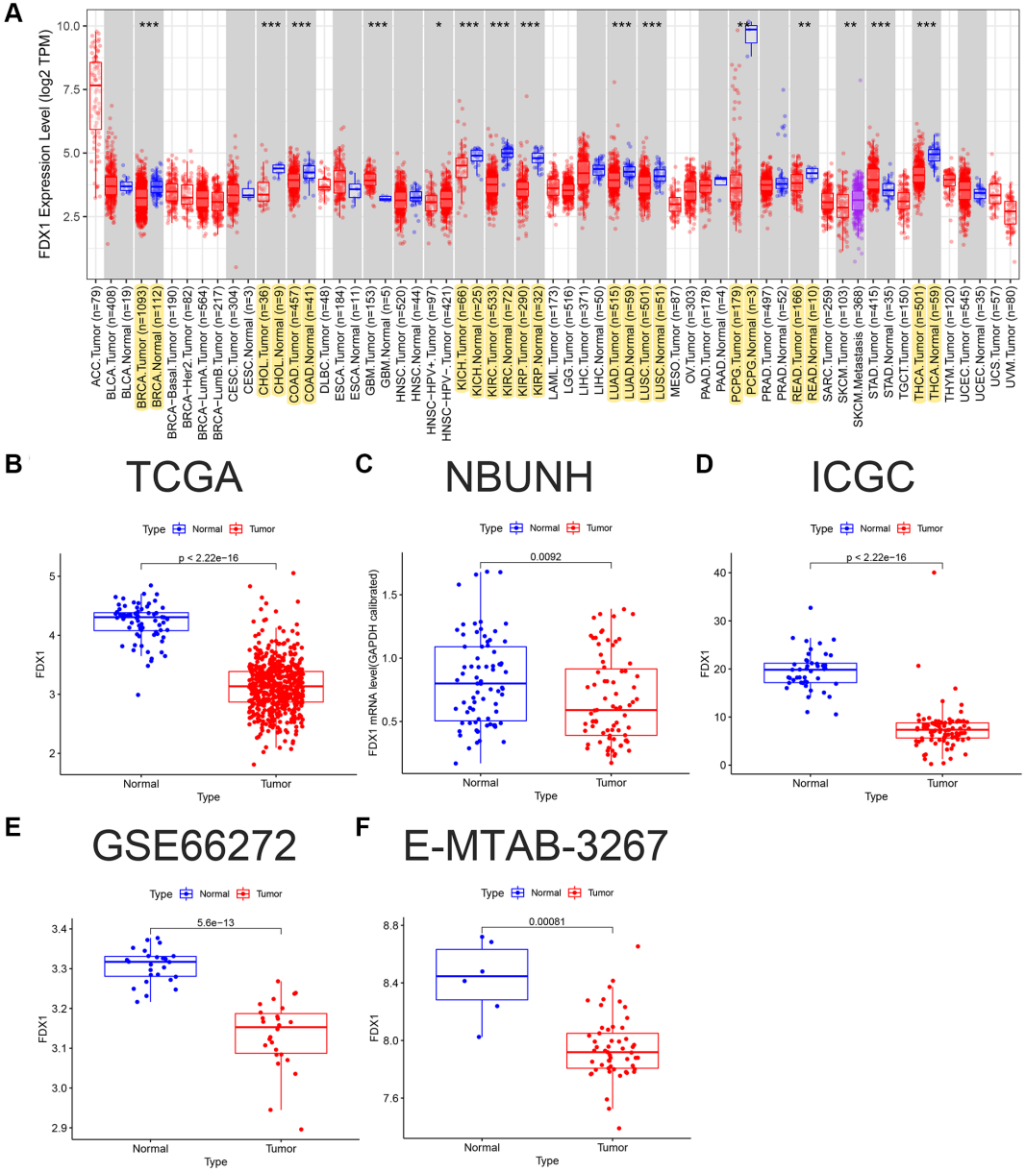
**FDX1 pan-carcinoma analysis and mRNA expression levels in ccRCC.** (**A**) Expression levels of FDX1 in pan-carcinoma from TCGA dataset. (**B**) Boxplot of FDX1 expression in TCGA dataset (KIRC) (*N* = 72, T = 539). (**C**) Boxplot of FDX1 expression in the NBUNH dataset (*N* = 75, T = 75). (**D**) Boxplot of FDX1 expression in the ICGC dataset (RECA-EU) (*N* = 45, T = 91). (**E**) Boxplot of FDX1 expression in the GEO dataset (GSE66272) (*N* = 25, T = 26). (**F**) Boxplot of FDX1 expression in ArrayExpress dataset (E-MTAB-3267) (*N* = 6, T = 53).

**Figure 2:**
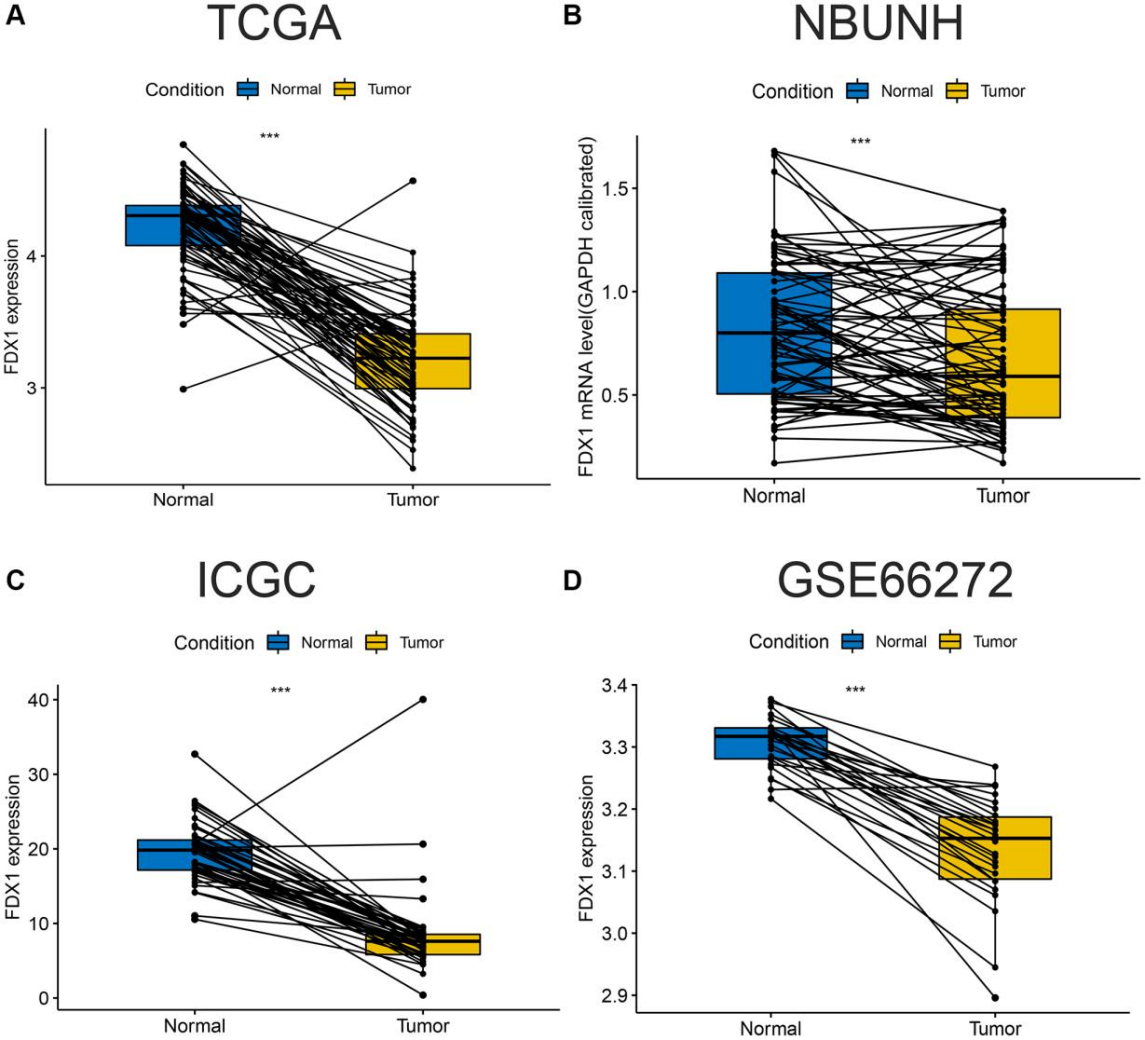
**Comparison of paired FDX1 mRNA expression levels in ccRCC.** (**A**) Boxplot of paired FDX1 expression levels in TCGA dataset (KIRC) (*N* = 72, T = 72). (**B**) Boxplot of paired FDX1 expression levels in the NBUNH dataset (*N* = 75, T = 75). (**C**) Boxplot of paired FDX1 expression levels in the ICGC dataset (RECA-EU) (*N* = 45, T = 45). (**D**) Boxplot of paired FDX1 expression levels in the GEO dataset (GSE66272) (*N* = 25, T = 25).

**Figure 3:**
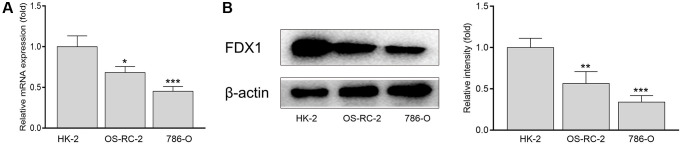
mRNA (**A**) and protein levels (**B**) of FDX1 in renal cancer cells and normal kidney cells. ^*^P < 0.05, ^**^P < 0.01 compared with the HK-2 group.

The expression of FDX1 in tumors was significantly lower than that in adjacent tumor tissues, including BRCA, CHOL, COAD, KICH, KIRC, KIRP, LUAD, LUSC, PCPG, READ, and THCA (Figure 1A). In addition, the expression of FDX1 in tumors was lower than that in adjacent tumor samples from TCGA (KIRC), International Cancer Genome Consortium (ICGC) (RECA-EU), Gene Expression Omnibus (GEO) (GSE66272), and ArrayExpress (E-MTAB-3267) databases (Figure 1B, 1D–1F). We also obtained the same results when comparing the same patient in pairs (Figure 2A, 2C, 2D). Furthermore, we found that the expression of FDX1 in adjacent tumor specimens was significantly higher than that in tumors after performing qRT-PCR in our independent clinical database (NBUNH), regardless of whether it was a pair or discrete (Figure 1C, 2B). In addition, compared with HK-2 cells, the mRNA and protein expression levels of FDX1 were lower in 786-O and OS-RC-2 cells (Figure 3A, 3B).

### Clinical analysis of FDX1

**Figure 4:**
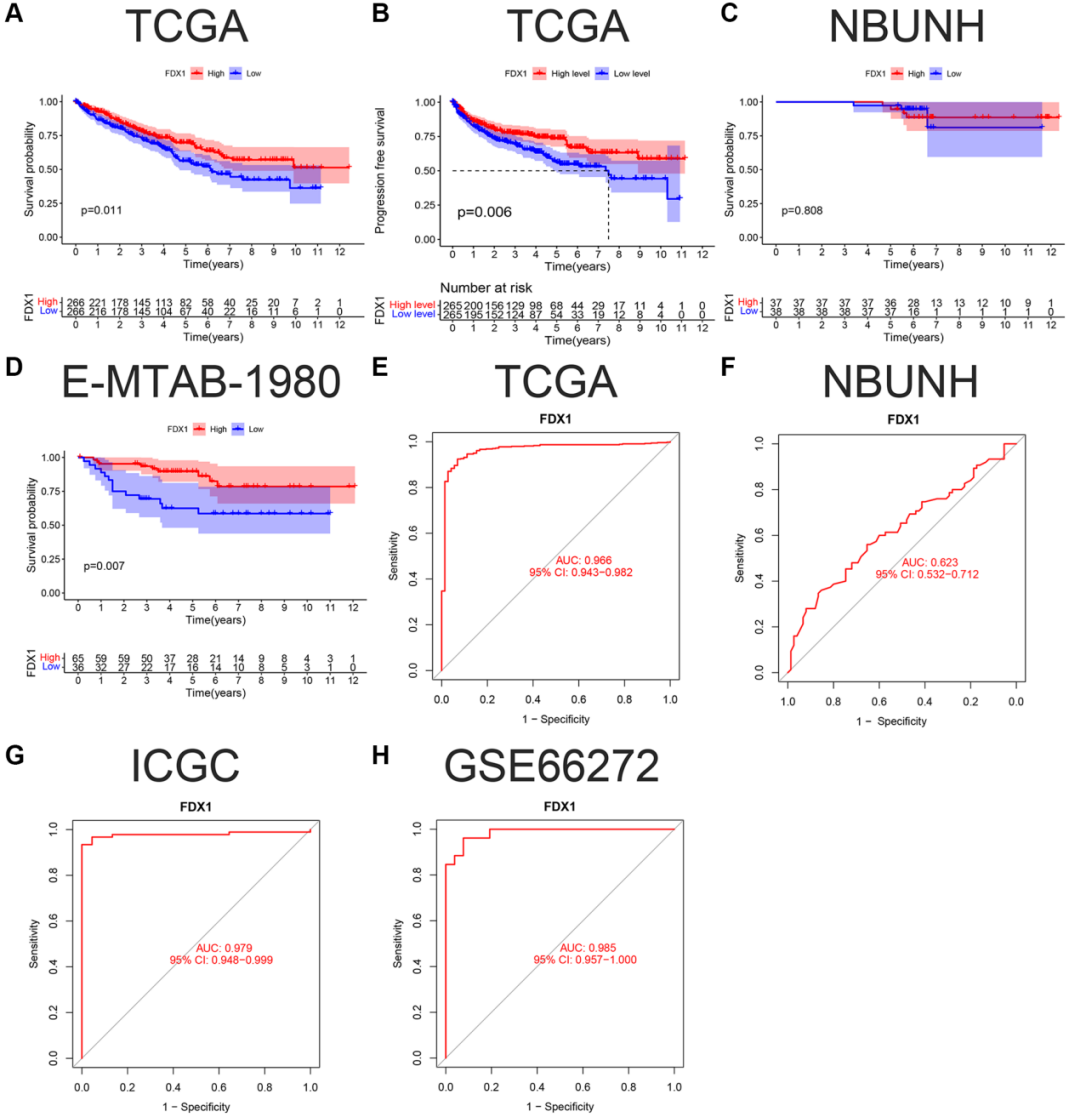
**K-M survival curve and ROC of FDX1 in ccRCC.** (**A**, **B**) OS (Overall survival) and DFS (Disease-free survival) in TCGA cohort. (**C**, **D**) OS in the NBUNH and E-MTAB-1980 cohorts. (**E**–**H**) ROC curves of TCGA, NBUNH, ICGC, and GSE66272 cohorts.

In this study, 532 patients with ccRCC were divided into high- and low-expression groups according to the median FDX1 expression level. Kaplan-Meier curves showed that the overall survival (OS) and progression free survival (PFS) in the low expression group were lower than those in the high expression group (*P* < 0.05, Figure 4A, 4B). However, there was no significant difference in the survival time in the NBUNH cohort (Figure 4C). In the E-MTAB-1980 dataset, when the best cutoff FDX1 expression level was selected, the OS in the low-expression group was significantly lower than that in the high-expression group (*P* < 0.01, Figure 4D). With the help of ROC (receiver operation curve) curves, we found that AUC (area under the curve) value of FDX1 was 0.966 (95% CI 0.943–0.982) in TCGA dataset (Figure 4E), 0.623 (95% CI 0.532–0.712) in the NBUNH cohort (Figure 4F), 0.979 (95% CI 0.948–0.999) in the ICGC dataset (Figure 4G), and 0.985 (95% CI 0.957–1.000) in the GEO dataset (Figure 4H).

**Figure 5:**
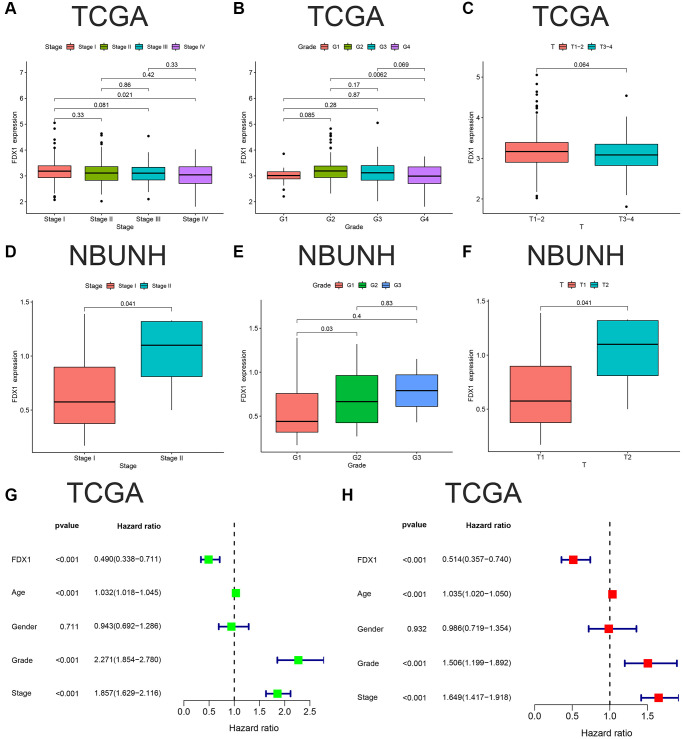
**Clinical correlation and independent prognostic analyses of FDX1.** (**A**–**C**) Associations between FDX1 and Stage, Grade, Stage T in TCGA database. (**D**–**F**) Association between FDX1 and Stage, Grade, Stage T in the NBUNH cohort. (**G**, **H**) Univariate and multivariate Cox regression analyses of clinicopathological variables and FDX1 in ccRCC.

The expression of FDX1 was lower in Stage IV than in Stage I in TCGA dataset (*P* < 0.05, Figure 5A), and the same was observed in grade 4 compared to grade 2 (*P* < 0.001, Figure 5B). There was no statistically significant difference between T1–2 and T3–4 (*P* > 0.05, Figure 5C). Our database (NBUNH) showed that the expression level of FDX1 in Stage II was higher than that in Stage I, G2 was higher than that in G1, and T2 was higher than that in T1 (all *P* < 0.05, Figure 5D–5F). The correlations between FDX1 expression levels and clinical characteristics from TCGA and NBUNH databases are shown in Table 1. Univariate and multivariate Cox analyses showed that FDX1 could be regarded as an independent prognostic indicator of OS in ccRCC (Figure 5G, 5H).

**Table 1:** Correlation between FDX1 expression and clinicopathological features in experimental (TCGA) and validation (NBUNH) cohorts.

**Characteristics/Cohorts**	**TCGA FDX1 expression**			**NBUNH FDX1 expression**		
	**Low (266)**	**High (266)**	***P* value**	**Low (38)**	**High (37)**	***P* value**
Age category			0.9714			0.0724
<65	172	161		29	21	
≥65	94	105		9	16	
Gender			0.0367	0.202
Male	184	161		17	22	
Female	82	105		21	15	
Vital status			0.0127			0.1884
Alive	165	192		35	33	
Dead	101	74		3	4	
Grade			0.0458			0.1942
G1	9	5		17	9	
G2	100	128		15	17	
G3	111	95		1	1	
G4	44	32		0	0	
NA	2	6		5	10	
Tumor stage			0.3682			0.1557
Stage I	122	144		37	33	
Stage II	31	26		1	4	
Stage III	66	57		0	0	
Stage IV	46	37		0		
NA	1	2		0	0	
T stage			0.3138			0.1557
T1	126	146		37	33	
T2	38	31		1	4	
T3	95	85		0		
T4	7	4		0		
M stage			0.3801			NA
M0	209	211		38	37	
M1	44	36		0	0	
NA	13	19		0	0	
N stage			0.1272			NA
N0	118	122		38	37	
N1	12	4		0	0	
NA	136	140		0	0	

NA: Clinical data are unknown.

### Function analyses of FDX1

**Figure 6:**
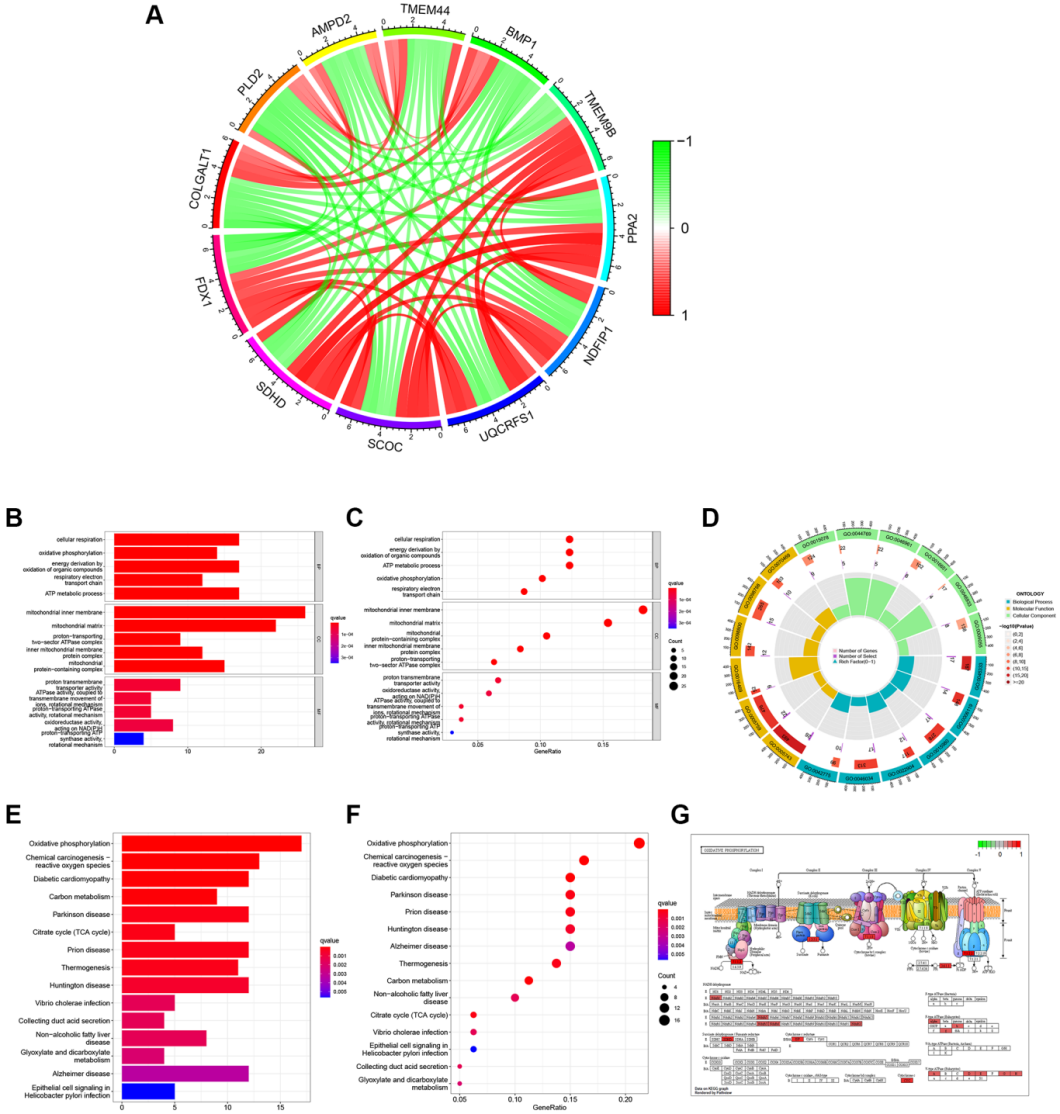
**Function and pathway analyses of FDX1 related genes.** (**A**) Circle graph of co-expression genes with FDX1 for GO functional analysis. Red represents a positive correlation and green represents a negative correlation. (**B**, **C**) Histogram and bubble diagram of GO. (**D**) Circle graph of GO. The first circle indicates 18 GO terms, with the coordinate scale of the gene number displayed outside the circle. The second circle shows the number of GO terms and Q values in the background gene. The third circle illustrates GO term number of associated genes. The fourth circle displays the abundance factor values for each GO term associated gene. (**E**, **F**) Histogram and bubble diagram in KEGG pathway analysis. (**G**) Pathway diagram of Oxidative Phosphorylation. Red background represents key genes with high expression.

There were 146 genes correlated to FDX1 considering the correlation coefficient >0.5 (Supplementary Table 1). The co-expression circle diagram shows the correlation between FDX1 and the other 11 genes with the largest absolute values of the correlation coefficient (Figure 6A). Gene Ontology (GO) categories included biological processes (BP), cellular components (CC), and molecular functions (MF) (Supplementary Table 2). We discovered that BP mainly contained cellular respiration, CC mainly included the mitochondrial inner membrane, and MF mainly contained the proton transmembrane (Figure 6B and 6C). The circular diagram depicts the top five GO functions (Figure 6D). Oxidative phosphorylation was closely associated with FDX1 expression in Kyoto Encyclopedia of Genes and Genomes (KEGG) (Figure 6E, 6F, Supplementary Table 3). Pathway graphs suggested that the genes correlated with the altered expression of oxidative phosphorylation (Figure 6G).

**Figure 7:**
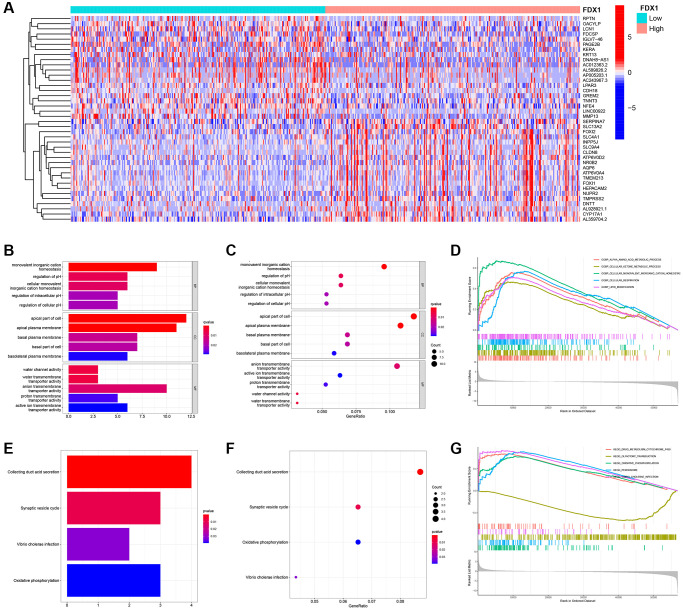
**Identification of differentially expressed genes, function, pathway, and GSEA analysis of related genes.** (**A**) Heat maps of the top 20 differential genes. (**B**, **C**) Histogram and bubble diagram of GO analysis. (**D**) GSEA enriches the functions of FDX1 in high- and low-expression groups. (**E**, **F**) Histogram and bubble diagram of KEGG analysis. (**G**) GSEA enriches the pathways of FDX1 in high- and low-expression groups.

Altogether, 234 differentially expressed genes (DEGs) were screened according to the cutoff values (Supplementary Table 4). A heatmap illustrating the expression of the top 20 DEGs is shown in Figure 7A. GO and KEGG analyses were performed to explore their functions (Supplementary Tables 5 and 6). We mainly found monovalent inorganic cation homeostasis in BP, the apical part of the cell in CC, and the anion transmembrane in MF (Figure 7B, 7C). On the other hand, collecting duct acid secretion and synaptic vesicle cycle of differential genes were enriched in KEGG pathways (Figure 7E, 7F), which was analyzed in “c5.go.v7.4. symbols.gmt” (Supplementary Table 7). The alpha amino acid metabolic process was enhanced when FDX1 was expressed at different levels (Figure 7D). Oxidative phosphorylation and the peroxisome were main pathways after GSEA analysis via “c2.cp.kegg.v7.4. symbols.gmt” (Figure 7G and Supplementary Table 8).

### FDX1 methylation, expression verification, and tumor mutational burden (TMB)

**Figure 8:**
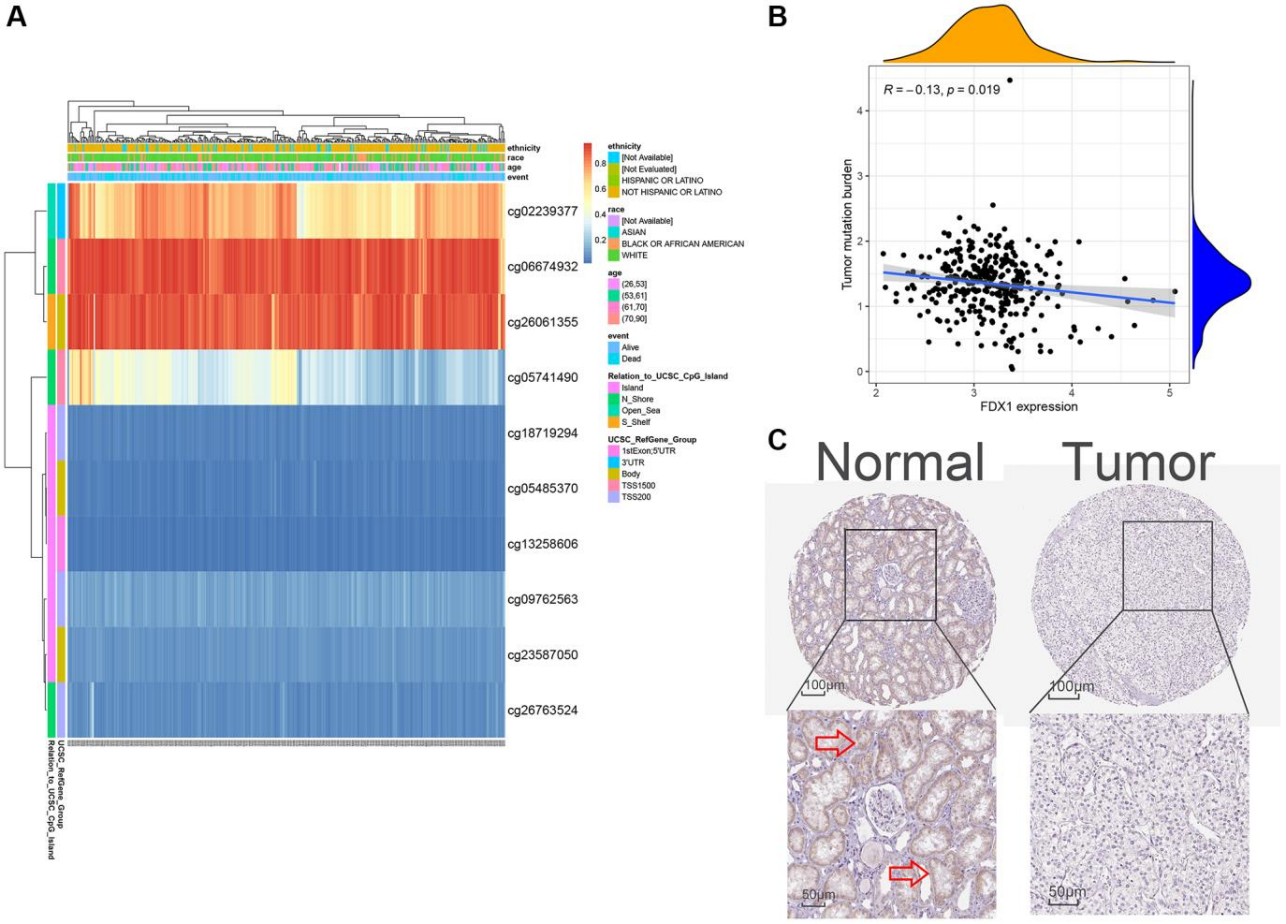
**Methylation, protein levels, and tumor mutational burden of FDX1.** (**A**) The heat map of DNA methylation of FDX1. (**B**) The tumor mutation burden of FDX1. (**C**) FDX1 protein levels based on HPA. The red arrow in C marks the site of FDX1 staining.

When mRNA is transcribed into a protein, it is modified by methylation in the transcription process. In the present study, FDX1 methylation was explored for its prognostic value in KIRC using MethSurv analysis. A DNA methylation heatmap illustrated that the highest FDX1 methylation level was in cg06674932 (Figure 8A). Overall, we identified eight CpGs in FDX1 that were significantly associated with ccRCC prognosis (Supplementary Table 9). TMB analysis demonstrated that FDX1 expression was negatively correlated with TMB levels (Spearman, R = −0.13, *P* = 0.019, Figure 8B). FDX1 was mainly expressed in the proximal and distal tubules of normal renal tissues but not in tumor tissues (Figure 8C).

### Immune infiltration and tumor microenvironment (TME) analyses of FDX1

**Figure 9:**
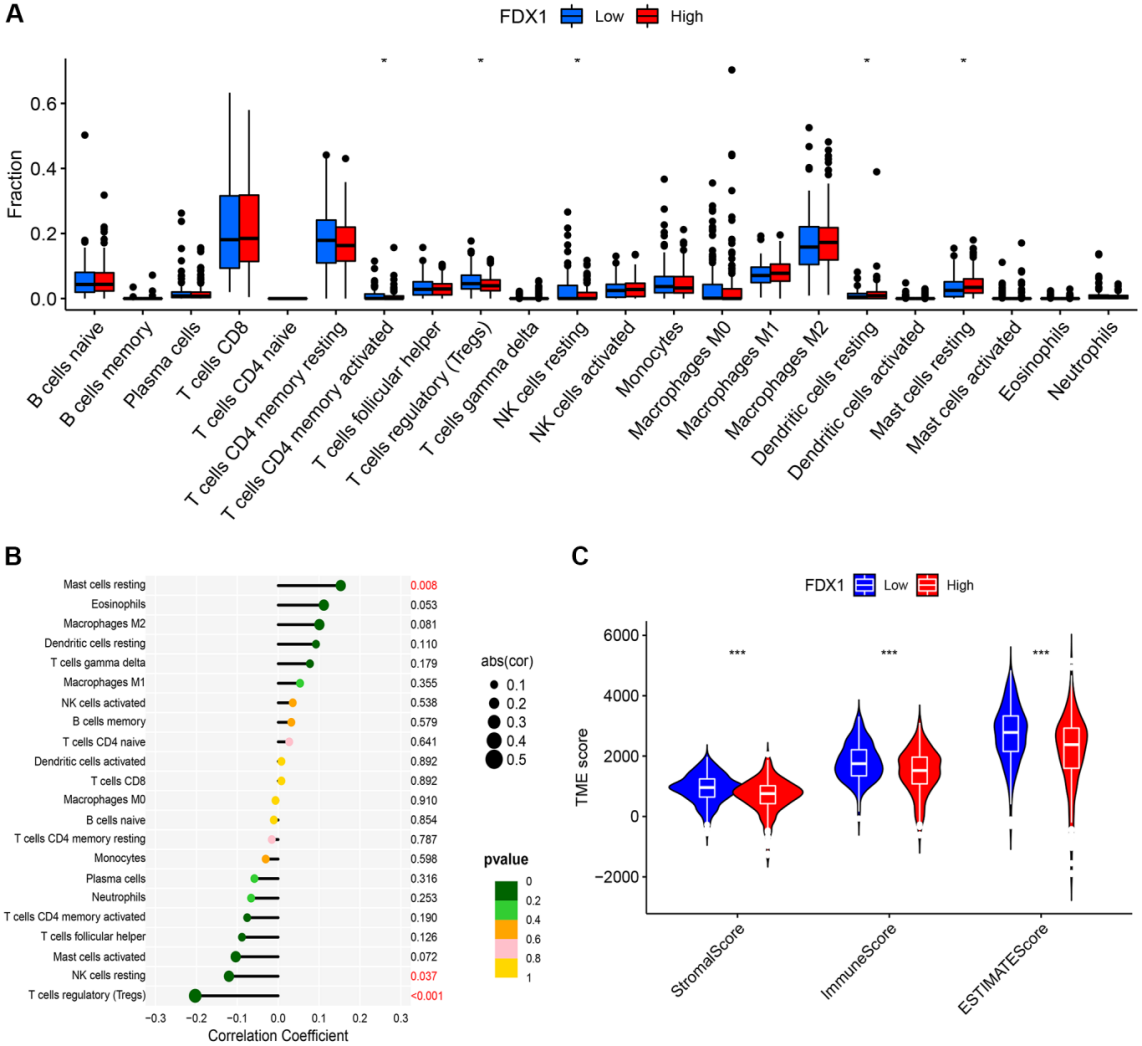
**Immune infiltration and immune microenvironment of FDX1.** (**A**) Boxplot showing the difference between the high- and low-expression of FDX1 groups referring to the proportion of 22 immune cells in KIRC tumor tissue. (**B**) Lollipop plot of correlation between FDX1 and 22 immune cells in TCGA cohort. (**C**) Violin graph showing the relationship between FDX1 expression and TME.

The CIBERSORT method explored 22 types of immune cells, of which five were found to be significantly different (*P* < 0.05, Figure 9A). In addition, correlation analysis showed that FDX1 was correlated with three types of immune cells (Figure 9B), suggesting that FDX1 could affect immune responses by regulating resting mast cells, resting NK cells, and regulatory T cells (Tregs). TME analysis indicated that stromal, immune, and ESTIMATE scores increased in the FDX1 low-expression group (*P* < 0.001; Figure 9C).

**Figure 10:**
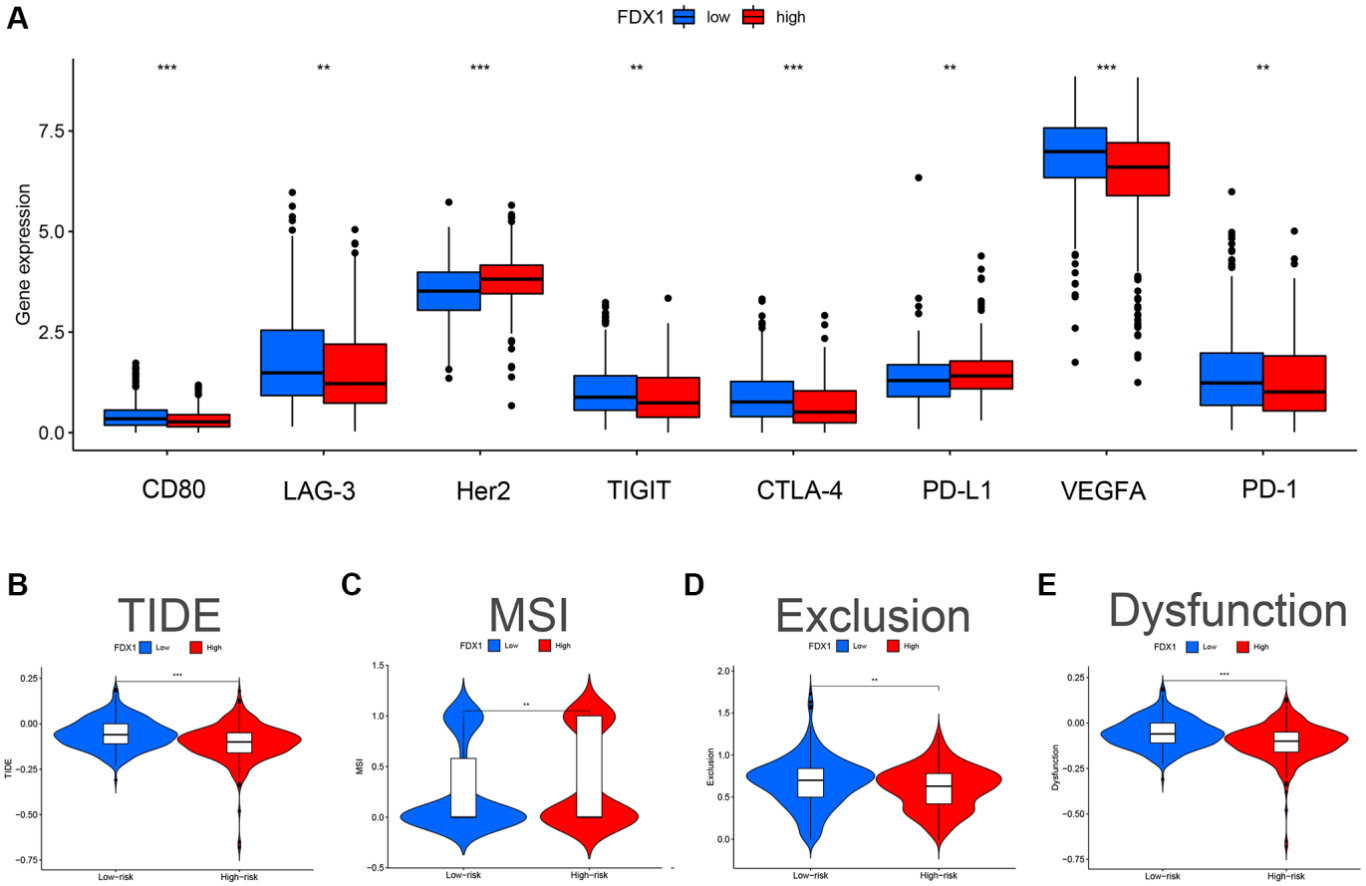
**Effect of FDX1 in high- and low-expression groups on TIDE and immunomodulatory target expression in ccRCC.** (**A**) Expression distribution of FDX1 at different immune checkpoints in TCGA database. (**B**) TIDE score of FDX1 high- and low-expression group. (**C**) MSI score of the FDX1 high- and low-expression group. (**D**) Exclusion score of FDX1 high- and low-expression group. (**E**) Dysfunction score of FDX1 high- and low-expression group.

We evaluated the differences in common immune cells (ICs) between the FDX1 high- and low-expression groups, including programmed cell death 1 (PD1/PDCD1), programmed cell death ligand 1 (PDL1/CD274) and cytotoxic T lymphocyte antigen 4 (CTLA4). Our results showed that most ICs were upregulated (Figure 10A) in the low-expression group. Furthermore, a higher Tumor Immune Dysfunction and Exclusion (TIDE) score was obtained in the low-expression group, demonstrating stronger immune dysfunction and immune resistance (Figure 10B–10E).

## DISCUSSION

Patients with ccRCC respond poorly to chemotherapy and radiotherapy [13]. Currently, ccRCC treatment is gradually transitioning to more accurate measures, such as targeted therapy and immune checkpoint inhibitors (ICIs) [14, 15]. In recent years, ICIs have changed the treatment pattern of advanced ccRCC and have gradually become the first-line treatment of choice [14, 16, 17]. However, reliable biomarkers are required to predict immune responses to ICIs. The metabolic characteristics of ccRCC are unique, with significant inhibition of glucose oxidation and activation of aerobic glycolysis [18]. FDX1 is an upstream regulator of protein lipid acylation and is a key gene that promotes copper sagging [18]. We hypothesized that FDX1 could serve as a vital biomarker for predicting ccRCC prognosis and reflecting immune responses to ICIs.

In this study, we determined the expression and transcriptional levels of FDX1 in ccRCC and discussed its main functions, particularly its important role in immunotherapy. The expression of FDX1 was decreased in ccRCC. After methylation, FDX1 is transcribed into a protein and expressed in normal tissues rather than in tumor samples. FDX1 regulates resting mast cells, resting NK cells, and regulatory T cells (Tregs), which influences the immune response in ccRCC. In addition, immune dysfunction and escape were observed in the low-expression group.

Multiple studies referencing TCGA and GEO databases have suggested decreased FDX1 expression in ccRCC tissues compared to that in normal tissues [12, 19]. In our study, we used an external database and qRT-PCR experiments for validation. The experimental results obtained by Zhang and Huang are consistent with our results [12, 19]. Our research also showed that FDX1 methylation could be observed during transcription, and the transcribed protein was expressed in normal tissues but not in tumors. This finding is supported by those of other studies [12, 19, 20]. Moreover, high FDX1 expression was associated with better OS according to TCGA and Array Express databases (*P* < 0.05).

However, our clinical cohort (NBUNH) showed no significant differences in FDX1 expression or OS. We propose that this phenomenon might be due to the well-taken measurements in the early screening and treatment of ccRCC or because many patients with advanced ccRCC prefer to seek hospitals in Shanghai and Beijing for surgery. We found that the patients who underwent surgery in our hospital were mainly those with early ccRCC. The decade of follow-up of these patients shows that they are still leading a high quality of life after their surgeries.

In addition, we analyzed the functions and pathways of FDX1-related genes and DEGs. Oxidative phosphorylation was found to be the most important pathway related to FDX1 in TCGA database, which was confirmed by KEGG and GSEA enrichment analyses. Similarly, some studies have found a close relationship between FDX1 and oxidative phosphorylation [10, 19, 21, 22]. As a tumor promoter, different levels of copper can regulate the oxidative phosphorylation of solid tumors, and when copper is exhausted, it can regulate mitochondrial oxidative phosphate to supplement the energy required by cancer cells [23, 24]. It was found that the deletion of SETD2 was related to the metabolic transformation to increase oxidative phosphorylation and lipogenesis in a ccRCC cell line [25]. Our pathway map also showed that SDHD, a gene correlated with FDX1, played a key role in oxidative phosphorylation. Cuproptosis is promoted by the inhibition of FDX1-mediated Fe-S cluster biosynthesis, with elesclomol specifically binding to FDX1 [26]. Thus, FDX1-related oxidative phosphorylation may be a novel target for cancer therapy [27].

Tumor cells can change the properties of the TME, thereby affecting its growth and spread. High TME scores were observed in patients with low FDX1 expression. The expression of FDX1 was positively correlated with resting mast cells and negatively correlated with resting NK cells and Tregs. Tregs can inhibit the proliferation and efficacy of CD8+T cells, which is considered one of the main obstacles to the successful clinical application of tumor immunotherapy [28]. FDX1 expression is also associated with the presence of several immune checkpoints such as CTLA4, PD-1, PD-L1, VEGFA, and LAG-3. The higher the predicted score of TIDE score, the greater the likelihood of immune evasion. And patients are less likely to benefit from immunotherapy. In our study, TIDE analysis showed that in the group with low FDX1 expression, poor response to ICIs might be related to higher TIDE scores, immune dysfunction, and immune exclusion. However, the mechanism underlying the relationship between FDX1 expression and ICI efficacy requires further investigation.

Although our results have been demonstrated to be reliable through a variety of verifications, including datasets from several external databases, protein expression levels, and basic experiments, there are some limitations. First, our 75 clinical specimens were all in an early stage, which does not reflect the advanced stage of ccRCC. Second, new therapeutic drugs referring to glycolysis inhibitors, such as elesclomol, require additional basic clinical trials to investigate their efficacy. Additional immunotherapy cohorts are required to validate and optimize our conclusions.

## CONCLUSIONS

We found that the expression of FDX1 was significantly downregulated in ccRCC at both mRNA and protein levels. Moreover, the expression of FDX1 was closely related to the clinicopathological features and prognosis of ccRCC. We found that FDX1 also plays an important role in oxidative phosphorylation. In summary, our study provides new insights into the relationships among copper death, metabolism, and immunity. However, additional basic research and multicenter cohort studies are required for further evaluation.

## MATERIALS AND METHODS

### Data collection and patient recruitment

RNA-seq and clinical information of patients with ccRCC were downloaded from TCGA database (KIRC; *N* = 72, T = 532; https://portal.gdc.cancer.gov/). In addition, the ICGC (RECA-EU; *N* = 45; T = 91; http://dcc.icgc.org), ArrayExpress (E-MTAB-1980; T = 101; E-MTAB-3267; *N* = 6, T = 53; https://www.ebi.ac.uk/arrayexpress/) and GEO (GSE66272; *N* = 25; T = 26; https://www.ncbi.nlm.nih.gov/geo/) databases were searched for external validation. All data were preliminarily processed using the “limma” R package. Details of the clinical information from each database are shown in Table 2.

**Table 2:** Summary of clinical characteristics of ccRCC patients.

**Characteristics/Datasets**	**TCGA *n* = 532**	**ICGC *n* = 91**	**E-MTAB-1990 *n* = 101**	**E-MTAB-3267 *n* = 53**	**GSE66272 *n* = 26**
Age category					
<65/≥65	333/199	57/34	52/49	38/15	13/13
Gender					
Male/Female	345/187	52/39	77/24	37/16	18/8
Vital status					
Alive/Dead	357/175	61/30	78/23	14/39	NA
Grade					
G1/G2/G3/G4/NA	14/228/206/76/8	NA	13/59/22/5/2	NA	1/16/8/1/0
Tumor stage					
I/II/III/IV/NA	266/57/123/83/3	NA	66/10/13/12/0	NA	12/1/12/1
T stage					
T1/T2/T3/T4/NA	272/69/180/11/0	NA	68/11/21/1/0	NA	NA
M stage					
M0/M1/MX	421/79/32	NA	89/12/0	NA	14/12/0
N stage					
N0/N1/N2/NA	240/16/0/276	NA	94/3/4/0	NA	NA

NA: Clinical data are unknown; *n*: The number of patients.

This study was approved by the Ethics Committee of the NBUNH and written informed consent was obtained from all included patients. A cohort of 76 tissue samples was collected from patients with ccRCC at the NBUNH. This cohort included patients with primary ccRCC who underwent radical nephrectomy in the Department of Urology since 2010. One sample was not used because its RNA was degraded. All clinical characteristics were obtained from the electronic information system of the hospital. Details included the initial age at diagnosis, sex, stage, and grade. Follow-up data were collected via telephone and Ningbo residents’ health records. Detailed information on the 75 ccRCC patients is shown in Supplementary Table 10.

### Western blot, qRT-PCR, and cell cultures

Cell lysates were harvested in RIPA buffer (Solarbio, Beijing, China) containing 1% PMSF protease inhibitor (Solarbio). Total protein concentration was calculated using a BCA protein assay kit (Beyotime, Beijing, China). Total protein (30 μg) samples were loaded and separated using 12% SDS-PAGE, transferred to PVDF membranes, blocked with 5% non-fat dry milk, and then incubated overnight with diluted primary antibody against FDX1 (Proteintech, Wuhan, China) or β-actin (Proteintech) at 4°C overnight. The blots were then washed with TBST, incubated with horseradish peroxidase-labeled secondary antibodies (Boster, Wuhan, China), and visualized using an enhanced chemiluminescence reagent.

Total RNA was extracted from the clinical samples and renal cancer cells using an RNA extraction kit (ServiceBio, Wuhan, China). The cDNA was synthesized according to the manufacturer’s instructions using a Servicebio^®^ RT First Strand cDNA Synthesis Kit (ServiceBio). The qRT-PCR used 2*SYBR Green qPCR Master Mix (ABclone, Woburn, MA, USA) for real-time quantitative PCR reaction. Overall, there were 40 cycles of denaturation at 95°C for 15 s and annealing/stretching at 60°C for 30 s. The following primers were used for qRT-PCR: FDX1, forward: 5′-CCACTTTATAAACCGTGATGGTG-3′; reverse: 5′-ACATGCACCAAAGCCATCAA-3′. GAPDH, forward: 5′-GGAAGCTTGTCATCAATGGAAATC-3′; reverse: 5′-TGATGACCCTTTTGGCTCCC-3′. GAPDH levels were used to standardize the data. The relative mRNA level of FDX1 was calculated by the 2^−ΔΔCt^ method.

The HK-2, OS-RC-2, and 786-O cell lines were purchased from the Cell Bank of the Chinese Academy of Sciences (Shanghai, China). HK-2 cells were cultured in DMEM (Hyclone, Logan, Utah, USA), and OS-RC-2 and 786-O cells were cultured in RMPI-1640 medium (Hyclone). All cells were incubated at 37°C in 5% CO_2_ after supplementing the culture medium with 10% heat-inactivated fetal bovine serum (Hyclone, Auckland, New Zealand), 100 U/mL streptomycin, and 100 mg/mL penicillin (Hyclone, Logan, UT, USA).

### Clinical survival analysis

TIMER 2.0 (http://timer.cistrome.org) was used to determine the expression levels of FDX1 in the tumors. The “ggplot2” and “ggpubr” R packages were applied to draw the box graph and compare the expression levels of FDX1 in tumor and adjacent normal samples. The R packages of “survival” and “survminer” were used to draw survival curves and analyze the different survival results in two groups with high- and low-expression levels according to the medium. The Receiver operating characteristic (ROC) curves were generated by the “pROC” R package, and the area under curve (AUC) values were calculated to evaluate the specificity and sensitivity of FDX1 in predicting benign and malignant tumors.

The clinical characteristics of the FDX1 high- and low-expression groups were compared in TCGA and NBUNH cohorts (details in Table 1). The clinicopathological parameters in high- and low-expression groups were compared using the “ggpubr” R package. Univariate and multivariate Cox regression analyses were performed using the Kaplan–Meier “survival” R package to assess the independence of FDX1 from other clinical factors.

### Analysis of the function and pathways

We screened out genes correlated with FDX1 using R software, meeting the requirements of |Pearson correlation coefficient|>0.5 and *P* < 0.001. The R packages “circlize” and “corrplot” were used to visualize the co-expression results. The R packages “clusterProfiler”, “org.Hs.eg.db”, “dplyr”, “enrichplot”, “ggplot2”, “circlize”, “RColorBrewer”, “ComplexHeatmap”, “R.utils”, and “pathview” were applied for GO and KEGG analyses. GO circle graphs and KEGG path diagrams were constructed.

DEGs in groups with high and low expression levels of FDX1 were screened using R software, meeting the criteria |logFC| >2 and FDR < 0.05. The R package “limma” was used to identify differential genes, and then “pheatmap” visualized the top 20 DEGs. GO and KEGG analyses of the DEGs were performed using the R packages “clusterProfiler”, “org.Hs.eg.db”, “enrichplot”, and “ggplot2.” GSEA enrichment analysis was done in MSigDB gene sets through “c5.go.v7.4. symbols.gmt” and “c2.cp.kegg.v7.4. symbols.gmt” in the R software.

### Methylation and TME analyses

MethSurv (https://biit.cs.ut.ee/methsurv/) was used to evaluate the prognostic value of FDX1 methylation in patients with ccRCC. The Protein Atlas Database (https://www.proteinatlas.org/) was used to display the protein expression level of FDX1. The TMB of FDX1 was calculated based on gene mutation data from TCGA.

The CIBERSORT algorithm revealed a relationship between FDX1 expression and 22 types of immune cells. The ESTIMATE algorithm was used to evaluate the immune microenvironment (ImmuneScore, robustness score, ESTIMATEScore, and TumorPurity) in groups with high and low FDX1 expression. The R packages “limma”, “estimate”, “e1071”, “reshape2”, “vioplot”, “ggExtra”, and “ggpubr” were used to complete the analyses. The box diagram and violin graph showed the results of immune cell infiltration and immune microenvironment. The correlation between FDX1 expression and 22 kinds of immune cells was illustrated by Lollipop graph.

The TIDE database was used to evaluate therapeutic effects. The expression matrix was uploaded to the website (http://tide.dfci.harvard.edu) to predict the possible immunotherapy effects with different expression levels of FDX1.

### Statistical analyses

A *t*-test was used to analyze the differences between groups of variables with a normal distribution. Otherwise, the Mann-Whitney *U* test was applied. A chi-square test was used for quantitative comparisons. The Pearson correlation method was used to analyze the correlation between two different genes. All statistical analyses were performed using the R software (v4.1.1) (https://www.r-project.org/). *P* < 0.05 was considered to be statistically significant. We marked ^*^ in the results, where ^*^ represents *P* < 0.05, ^**^ represents *P* < 0.01, and ^***^ represents *P* < 0.001.

### Institutional review board statement

The study was conducted in accordance with the Declaration of Helsinki and approved by the Institutional Ethics Committee of Ningbo Urology and Nephrology Hospital.

### Data availability statement

The data supporting the findings of this study are available from KIRC at https://portal.gdc.cancer.gov/, ICGC at http://dcc.icgc.org, ArrayExpress at https://www.ebi.ac.uk/arrayexpress/, and GEO at https://www.ncbi.nlm.nih.gov/geo/. The authors confirm that the data supporting the findings of this study are available in the article and supplementary material.

## Supplementary Materials

Supplementary Tables 1-5, 7-8 and 10

Supplementary Tables 6 and 9
