# Supplementary material for: Confirmation of the predictive function of cuproptosis-related gene FDX1 in clear cell renal carcinoma using qRT-PCR and western blotting
Source: Aging (Albany NY). 2023 Jul 10;15(13):6117–34. doi: 10.18632/aging.204807 (PMC10373983; doi:10.18632/aging.204807)
Supplement: Supplementary Tables 6 and 9 [file aging-15-204807-s002.pdf]

SUPPLEMENTARY TABLES

Supplementary Table 6. KEGG analysis of the differential genes

ID	Description	GeneRatio	BgRatio	*p*-value	p.adjust	*q*-value	geneID	Count
hsa04966	Collecting duct acid secretion	4/46	27/8163	1.41E-05	0.001239892	0.001239892	ATP6V0D2/ATP4B/ATP6V0A4/SLC4A1	4
hsa04721	Synaptic vesicle cycle	3/46	78/8163	0.009487054	0.417430362	0.417430362	ATP6V0D2/SLC18A3/ATP6V0A4	3
hsa05110	Vibrio cholerae infection	2/46	50/8163	0.032066051	0.664508171	0.664508171	ATP6V0D2/ATP6V0A4	2
hsa00190	Oxidative phosphorylation	3/46	134/8163	0.039335197	0.664508171	0.664508171	ATP6V0D2/ATP4B/ATP6V0A4	3

Supplementary Table 9. 8 CpGs in FDX1 associated with ccRCC prognosis.

Gene	CpG	HR	LR Test *P* value
FDX1	Body-Island-cg05485370	0.518	0.0069
FDX1	Body-Island-cg23587050	0.408	1.70E-05
FDX1	TSS200-Island-cg09762563	1.721	0.0078
FDX1	1stExon;5'UTR-Island-cg13258606	0.395	9.20E-05
FDX1	TSS1500-N_Shore-cg05741490	2.106	0.0036
FDX1	TSS1500-N_Shore-cg06674932	1.996	0.006
FDX1	TSS200-N_Shore-cg26763524	1.981	0.0011
FDX1	Body-S_Shelf-cg26061355	0.573	0.0098
